# Bypass of *Candida albicans* Filamentation/Biofilm Regulators through Diminished Expression of Protein Kinase Cak1

**DOI:** 10.1371/journal.pgen.1006487

**Published:** 2016-12-09

**Authors:** Carol A. Woolford, Katherine Lagree, Wenjie Xu, Tatyana Aleynikov, Hema Adhikari, Hiram Sanchez, Paul J. Cullen, Frederick Lanni, David R. Andes, Aaron P. Mitchell

**Affiliations:** 1 Department of Biological Sciences, Carnegie Mellon University, Pittsburgh, Pennsylvania, United States of America; 2 Department of Biological Sciences at the University at Buffalo, Buffalo, New York, United States of America; 3 Departments of Medicine and Medical Microbiology and Immunology, University of Wisconsin, Madison, Wisconsin, United States of America; Duke University Medical Center, UNITED STATES

## Abstract

Biofilm formation on implanted medical devices is a major source of lethal invasive infection by *Candida albicans*. Filamentous growth of this fungus is tied to biofilm formation because many filamentation-associated genes are required for surface adherence. Cell cycle or cell growth defects can induce filamentation, but we have limited information about the coupling between filamentation and filamentation-associated gene expression after cell cycle/cell growth inhibition. Here we identified the CDK activating protein kinase Cak1 as a determinant of filamentation and filamentation-associated gene expression through a screen of mutations that diminish expression of protein kinase-related genes implicated in cell cycle/cell growth control. A *cak1* diminished expression (DX) strain displays filamentous growth and expresses filamentation-associated genes in the absence of typical inducing signals. In a wild-type background, expression of filamentation-associated genes depends upon the transcription factors Bcr1, Brg1, Efg1, Tec1, and Ume6. In the *cak1* DX background, the dependence of filamentation-associated gene expression on each transcription factor is substantially relieved. The unexpected bypass of filamentation-associated gene expression activators has the functional consequence of enabling biofilm formation in the absence of Bcr1, Brg1, Tec1, Ume6, or in the absence of both Brg1 and Ume6. It also enables filamentous cell morphogenesis, though not biofilm formation, in the absence of Efg1. Because these transcription factors are known to have shared target genes, we suggest that cell cycle/cell growth limitation leads to activation of several transcription factors, thus relieving dependence on any one.

## Author Summary

The ability of the pathogen *Candida albicans* to grow on surfaces as biofilms is a determinant of infection ability, because biofilms on implanted medical devices seed infections. Biofilm formation by this organism requires growth in the form of filamentous cells and the expression of filamentation-associated genes. Inhibition of cell proliferation can induce filamentous cell formation, as we find here for strains that express greatly reduced levels of the cell cycle regulator Cak1. Surprisingly, biofilm formation occurs independently of many central biofilm regulatory genes when Cak1 levels are reduced. This response to proliferation inhibition may reflect the activation of numerous biofilm regulators, thus relieving the dependence on any one regulator. The stimulation of biofilm formation by proliferation inhibition, a property of many bacterial pathogens as well, may contribute to the limited effectiveness of antimicrobials against biofilms.

## Introduction

*Candida albicans* is an invasive fungal pathogen that causes lethal infections in approximately 400,000 people per year worldwide [1]. Susceptibility to infection can be caused by a weakened immune system or presence of an implanted medical device, which provides a niche for biofilm formation. Limitations of the antifungal armamentarium lead to a 40% mortality rate among infected patients despite therapy [2–4]; hence the development of new therapeutics is of the utmost importance [5].

The goal of our study is to tie essential genes, which are candidate drug targets [6–9], to biological processes. Such connections may be useful to develop screens for growth inhibitors. Several approaches have been used with *C*. *albicans* to reduce expression of an essential gene in order to assess its biological function [10–17]. Most approaches have used promoters with activity that can be regulated by presence of a nutrient or small molecule (Tet-off [12–14], *MET3* [15], *PCK1* [16], and *MAL2* [17]). In fact, a collection of GRACE strains is now available in which 2,356 different genes have been placed under control of a doxycycline-repressible promoter [12, 18]. This approach allows growth under a permissive condition in which the gene of interest is expressed at high levels, and then allows functional assays after expression of the gene is reduced.

Several previous studies of *C*. *albicans* have shown that cell cycle or cell growth inhibition induces polarized growth, yielding elongated cells that resemble naturally occurring filamentous cells such as hyphae or pseudohyphae (reviewed in [19, 20]; see [18] for a recent study). Filamentous growth is of particular significance for *C*. *albicans* because it is required for invasive infection and for biofilm formation.

Many filamentous cell functions are tied to their expression of a distinctive set of filamentation-associated genes whose products have direct roles in adherence and pathogenicity [21, 22]. It is not clear to what extent cell cycle/cell growth inhibition induces filamentation-associated gene expression, as illustrated by the foundational study of Bachewich et al. [23]. They used genome-wide profiling to establish that depletion of the essential protein kinase Cdc5, an M-phase regulator, induced both filamentous growth and several filamentation-associated genes that included *ECE1* and *RBT1*. However, they found that filamentous growth and filamentation-associated gene expression could be uncoupled. Specifically, treatment with the DNA synthesis inhibitor hydroxyurea induced filamentous growth but had little effect on filamentation-associated gene expression. A more extreme case of uncoupling comes from work of Bastidas et al. [24] who studied cell growth control by the Tor1 kinase. They observed that the Tor1 inhibitor rapamycin blocked filamentous growth yet induced filamentation-associated genes that included *ECE1* and *RBT1*. Therefore, the extent of coupling between filamentation and filamentation-associated gene expression after cell cycle/cell growth inhibition is uncertain. In addition, the identities of transcriptional regulators that mediate the effects of cell cycle/cell growth inhibition on filamentation-associated genes remain largely undefined.

We focus here on a set of protein kinase genes that have been implicated in cell cycle or cell growth control. We chose to examine protein kinases because they are among the most druggable eukaryotic protein targets [25, 26]. In fact, our prior study of *C*. *albicans* protein kinase-related gene insertion mutants revealed that many non-essential protein kinases may be useful antimicrobial targets [27]: 35 of 80 viable mutants were defective in virulence-associated traits that included stress tolerance, biofilm formation, or filamentation. However, because DNA insertions were used to create the mutations in that study, we were unable to recover mutations in essential protein kinase genes. Here we have engineered constitutive diminished expression of 15 protein kinase and protein kinase-related genes. Our findings extend the connection between cell cycle/cell growth inhibition and filamentation to include filamentation-associated gene expression. We find that the impact of a partial defect in cell cycle regulator Cak1 is sufficient to promote biofilm formation and filamentation-associated gene expression in the absence of major transcriptional activators of these processes.

## Results

### Construction of DX strains

We chose 18 protein kinase genes that we had been unable to disrupt previously [27] for functional analysis (Table 1). Half of the genes had been included in a screen of doxycycline-repressible GRACE strains for altered hyphal morphogenesis [12]; the other half were not represented among the GRACE strains (Table 1). In addition, we included as an internal control an essential protein kinase-related gene, *CLN3*, that had been analyzed previously through conditional expression approaches [28, 29].

**Table 1:** DX mutants in protein kinase-related genes.

ORF	Gene name or alias	*UAU1* insertion isolated[27]	Null or repressible allele	DX mutant isolated	GRACE strain reported[18]	DX strain morphology^a^	Depletion strain morphology	DX gene expression (P-value)^b^
ORF19.793	*CAK1*	no	repressible[18]	yes	yes	filamentous	yeast[18]	120 (0.03)
ORF19.1960	*CLN3*		repressible[18, 28, 29]	yes	yes	filamentous	filamentous[28, 29]	171 (0.03)
ORF19.1619	*CTK1*	no		yes	no	filamentous		15 (0.03)
ORF19.1223	*DBF2*	no	repressible[30]	yes	no	filamentous	yeast[30]	13 (0.19)
ORF19.663	*GIN4*	no	null[31]	yes	no	filamentous	filamentous[31]	12 (0.03)
ORF19.3474	*IPL1*	no	repressible[18]	yes	yes	filamentous	filamentous[18]	4 (0.19)
ORF19.6239	*KIN28*	no	repressible[18]	yes	yes	filamentous	filamentous[18]	46 (0.03)
ORF19.1936	*SNF1*	no	repressible[18]	yes	yes	filamentous	filamentous[18]	4 (0.5)
ORF19.3459	*MCK1*	no		yes	no	filamentous		1 (0.5)
ORF19.3561	*CDC7*	no	repressible[18]	yes	yes	yeast	filamentous[18]	2 (0.19)
ORF19.3856	*CDC28*	no	repressible[18, 32]	yes	yes	yeast	filamentous[18]	1 (0.19)
ORF19.1754	*CMK2*	no	null[33, 34]	yes	no	yeast	yeast[33, 34]	1 (0.8)
ORF19.5068	*IRE1*	no	insertion[27]	yes	no	yeast		1 (0.8)
ORF19.3840	*SAK1*	no		yes	no	yeast		1 (0.19)
ORF19.3669	*SHA3*	no	repressible[18]	yes	yes	yeast	yeast[18]	2 (0.03)
ORF19.6010	*CDC5*	no	repressible[35]	no	yes		filamentous[35]	
ORF19.7293	*MPS1*	no	repressible[18, 36]	no	yes		filamentous[18]	
ORF19.6846	*PHO85*	no	repressible[37]	no	no		yeast[37]	
ORF19.2320	*RIO1*	no		no	no			

^a^ Based on Fig 1

^b^ Gene expression values are the mean fold increase in RNA levels for five core filamentation genes (Fig 1) in the DX strain compared to the wild-type strain. Statistical significance was assessed by a sign test.

To create a set of strains with decreased expression of each gene, we deleted one allele and replaced the 5' region of the second allele with a weakly expressed promoter. We were able to introduce the *PGA5* promoter in front of most genes to create *DX1* alleles (S1 Table). However, for three genes, only fusions to the promoters of *PGA42* (*DX2* alleles) or *ORF19*.*7606* (*DX3* alleles) were recovered (S1 Table). We refer to a strain of genotype *yfg1-DX1/yfg1Δ*, *yfg1-DX2/yfg1Δ*, or *yfg1-DX3/yfg1Δ* as a *yfg1* DX strain. Of the 19 genes chosen (18 protein kinase genes and *CLN3*), we recovered DX strains for 15 genes (Table 1).

We used nanostring RNA measurements to determine whether the DX strains had reduced expression of the targeted genes (S1 Table). Under yeast growth conditions (30° in YPD medium), the strains displayed 7.3 ± 8.1% of the wild-type expression levels for the 12 genes with *DX1* alleles. Under hyphal growth conditions (37° in YPD + serum medium), the strains displayed 8.5 ± 10.8% of the wild-type expression levels for the 12 genes with *DX1* alleles. Too few genes were represented by *DX2* or *DX3* alleles to draw general conclusions. These results indicate that DX strains engineered by promoter replacement with the *PGA5* promoter have reduced expression of several different targeted genes under both yeast and hyphal growth conditions.

### DX strain filamentation

**Fig 1:**
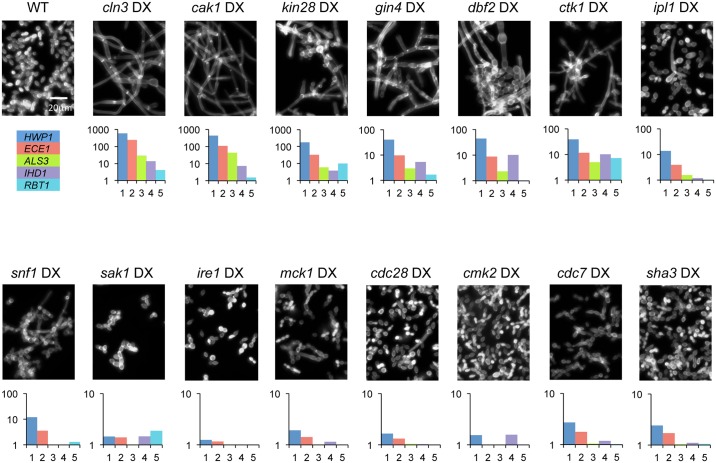
DX mutant phenotypes. Strains were stained with Calcofluor White after 4 hr growth at 30°C in YPD medium. Expression levels of core filamentation genes in mutants grown for 4 hr at 30° in YPD are expressed as fold-change to the wild type; complete data are in S2 Table. Strains: WT (DAY286), *cln3* DX (CW994), *cak1* DX (CW1003), *kin28* DX (CW1041), *gin4* DX (CW900), *dbf2* DX (CW914), *ctk1* DX (CW1005), *ipl1* DX (CW1038), *snf1* DX (CW927), *sak1* DX (CW995), *ire1* DX (CW906), *mck1* DX (CW1006), *cdc28* DX (CW991), *cmk2* DX (CW999), *cdc7* DX (CW993), *sha3* DX (CW1010).

Several DX strains grew as filamentous forms under conditions that supported nonfilamentous growth of the wild-type strain (Fig 1, Table 1). The *cln3*, *cak1*, *kin28*, *gin4*, *dbf2*, and *ctk1* mutants had the most extreme phenotypes; the *snf1* and *mck1* mutants had prominent though less severe phenotypes. Many of the remaining mutants had mild or heterogeneous phenotypes (summarized in Table 1). Filamentous cells of the *cln3* and *cak1* DX strains resembled true hyphae in having parallel cell walls along the filament (Fig 1). For many of the other mutants, filament diameters varied and lengths were stunted (see especially *kin28*, *gin4*, *dbf2*, *ctk1*, and *snf1*). Our observations with the *cln3* DX strain agreed with previous studies of hyperpolarized growth after repression of *CLN3* RNA [28, 29].

Filamentous growth of wild-type *C*. *albicans* is accompanied by high-level expression of filamentation-associated genes [19, 38]. Therefore, we sought to determine whether filamentous growth of DX strains observed under noninducing conditions was accompanied by filamentation-associated gene expression. We measured expression of 181 genes in each DX mutant, including five of the eight core filamentation genes [38]: *HWP1*, *ECE1*, *ALS3*, *IHD1*, and *RBT5*. Many of the mutants display elevated expression of these core filamentation genes (Fig 1, Table 1). In general, the extent of core filamentation gene up-regulation correlated with the degree of hyperpolarized growth: *cln3*, *cak1* and *kin28* DX strains expressed the highest levels of *HWP1*, *ECE1* and *ALS3*, followed by *gin4* and *ctk1* DX strains. These results argue that growth limitation imposed by reduced expression of four protein kinase genes, or the essential protein kinase-related gene *CLN3*, activates both morphogenesis and gene expression arms of the filamentous growth program.

Several DX strains provided exceptions to correlation between filamentous morphology and filamentation gene expression. This group included *dbf2*, *ipl1*, *snf1*, and *ire1* DX mutants. These strains displayed filamentous morphology and seemed to have slightly elevated core filamentation gene expression, but the difference from the wild type was not statistically significant (Table 1). We believe that the poor correlation between morphology and gene expression in this group reflects the small number of core filamentation genes. In fact, a large number of other genes that were up-regulated in this set of DX mutants (as well as the *cln3*, *cak1*, *kin28*, *gin4*, and *ctk1* DX mutants) under noninducing conditions (YPD, 30°) were also up-regulated in the wild-type strain under filamentation-inducing conditions (YPD + 10% serum, 37°) (S1 Fig; S2 Table). An additional complication was that several of these DX mutants had heterogeneous morphology (Fig 1), so the gene expression profile of the population may not reflect the gene expression profile of the rare filamentous cells. In summary, there is a good correlation between filamentation and core filamentation gene expression for DX mutants with the most severe phenotypes, and an uncertain correlation for DX mutants with weak or heterogeneous phenotypes.

### DX mutant validation

**Fig 2:**
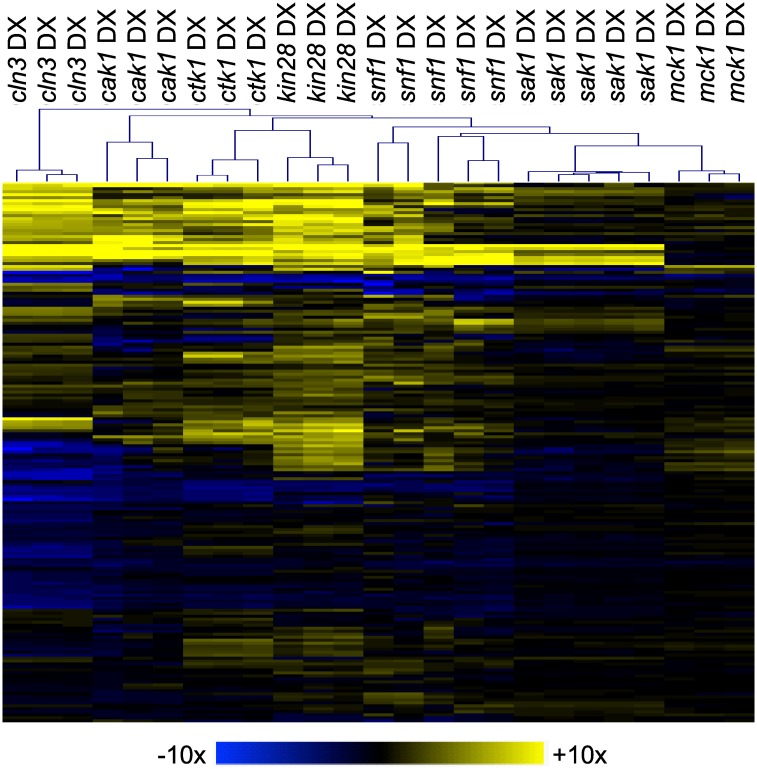
DX mutant isolate comparison. Multiple isolates of each DX strain indicated were obtained from a single transformation of a heterozygous deletion mutant. RNA was extracted from cells grown for 4 hr at 30°C in YPD and used for nanoString expression analysis (S3 Table). Hierarchal clustering of gene expression data was performed using MeV software. Fold change values were obtained by dividing normalized expression values for each mutant strain by the wild-type strain (DAY185) for each of the probes. The color scale represents Log2 fold change compared to wild type. (Blue limit: 10-fold down; yellow limit: 10-fold up.) Strains are listed in S3 Table.

We sought to verify that each defined mutation was the cause of each mutant strain's phenotype. We took two approaches to establish a genotype-phenotype correlation: independent isolate characterization and complementation. For the first approach, we chose seven genes and characterized 3–5 five mutant isolates of each: *cln3*, *cak1*, *ctk1*, *kin28*, *snf1*, *sak1* and *mck1*. For the 5 genes that caused hyperfilamentous morphology, we observed that all independent isolates were hyperfilamentous, as indicated by wrinkled colony morphology [39]. In addition, independent DX isolates for most genes showed highly reproducible expression alterations for 181 genes assayed (Fig 2, S3 Table). The one exception was that independent *snf1* DX strains had a more variable gene expression phenotype than the other DX strains. It is possible that the *snf1* DX strains acquire second-site suppressor mutations that have impact on their gene expression phenotype. These results indicate that independent DX isolates for six of the seven genes tested yield reproducible phenotypes, and support the argument that the defined DX mutations cause the mutant phenotypes in those cases.

**Fig 3:**
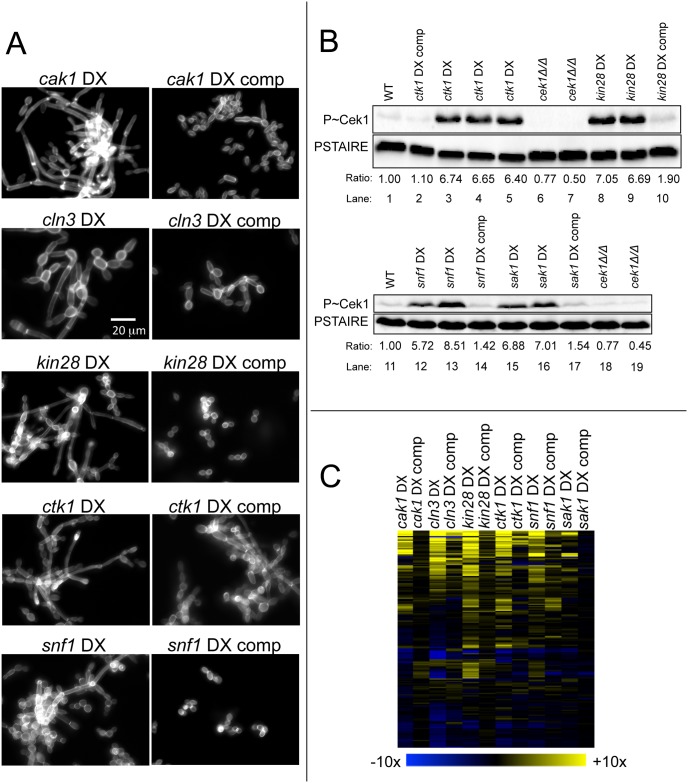
DX mutant complementation. A. Cell morphology. Strains were stained with Calcofluor White after 4 hr growth at 30°C in YPD medium. Strains: *cak1* DX (CW1428), *cak1* DX comp (CW1431), *cln3* DX (CW1437), *cln3* DX comp (CW1440), *kin28* DX (CW1082), *kin28* DX comp (CW1085), *ctk1* DX (CW1076), *ctk1* DX comp (CW1078), *snf1* DX (CW1138), *snf1* DX comp (CW1141). B. Cek1 phosphorylation. Immunoblots were performed on yeast extracts from mid-log phase cells grown for 5 hr at 30°C. Strains for lanes 1, 3, 6, 8, 11, 12, 15, and 18 are His^-^; strains for lanes 2, 4, 5, 7, 9, 10, 13, 14, 16, 17, and 19 are His^+^. The lanes show experiments with the strain in parentheses: 1 (DAY286), 2 (CW1078), 3 (CW1005), 4 (CW1075), 5 (CW1076), 6 (TA72), 7 (TA86), 8 (CW1041), 9 (CW1082), 10 (CW1085), 11 (DAY286), 12 (CW927), 13 (CW1138), 14 (CW1141), 15 (CW995), 16 (CW1111), 17 (CW1114), 18 (TA72), 19 (TA86). C. Gene expression profiles. RNA was extracted from cells grown for 4 hr at 30°C in YPD and used for nanoString expression analysis (S3 Table). Hierarchal clustering of gene expression data was performed using MeV software. Fold change values were obtained by dividing normalized expression values for each mutant or complemented strain by the wild type strain (DAY185) for each of the probes. The color scale represents Log2 fold change compared to wild type. (Blue limit: 10-fold down; yellow limit: 10-fold up.) Strains are listed in S3 Table.

The second approach to test the genotype-phenotype relationship was complementation. We introduced a wild-type copy of the respective affected gene into each of six DX mutants: *cak1*, *cln3*, *kin28*, *ctk1*, *snf1*, and *sak1*. Complementation was assessed through cell morphology, Cek1 phosphorylation, and gene expression profiling. Five of the DX mutants in this group were hyperfilamentous, and we observed that the complemented *cln3*, *kin28*, *cak1*, *ctk1*, and *snf1* DX strains had nonfilamentous morphology when grown at 30° (Fig 3A), similar to the wild-type strain (Fig 1). The *sak1* DX strain had nonfilamentous morphology under these conditions, so complementation could not be assessed by morphology. These results indicate that reduced expression of *CLN3*, *KIN28*, *CAK1*, *CTK1*, or *SNF1* causes hyperfilamentation.

Complementation was assessed through levels of phosphorylated MAP Kinase Cek1 as well. Activity of Cek1 has been implicated in both filamentation and growth ([40–42]; reviewed in [43]). Immunoblot analysis revealed that the *ctk1*, *kin28*, *snf1*, and *sak1* DX mutants had increased levels of phosphorylated Cek1 compared to the wild type (Fig 3B). Complementation of these DX mutants reduced levels of phosphorylated Cek1 (Fig 3B). These results indicate that reduced expression of *CTK1*, *KIN28*, *SNF1*, or *SAK1* causes increased accumulation of phosphorylated Cek1.

Complementation was also assessed through assays of RNA levels for 181 genes (Fig 3C, S3 Table). We observed that the most prominent gene expression changes were almost completely reversed by complementation for most of the DX mutants tested. Specifically, the mean fold-change in expression for the 20 most greatly affected genes, compared to the wild-type strain, was reduced in the complemented strains from 9.4 to 1.2 (*cak1)*, 10.1 to 1.5 *(kin28)*, 8.4 to 2.0 (*ctk1*), 6.3 to 1.5 (*snf1*), and 2.9 to 1.1 *(sak1)*. We observed partial complementation of the *cln3* DX mutant: introduction of wild-type *CLN3* reduced the mean fold-change in expression for the 20 most greatly affected genes from 12.6 to 3.1. The level of *CLN3* RNA in the complemented strain was ~20% of the wild-type level (S3 Table), which may be the reason for partial complementation. Our analysis supports the idea that reduced expression of *CAK1*, *CLN3*, *KIN28*, *CTK1*, *SNF1*, and *SAK1* causes the major gene expression changes observed in the respective DX strains.

### Cak1 function in biofilm formation

**Fig 4:**
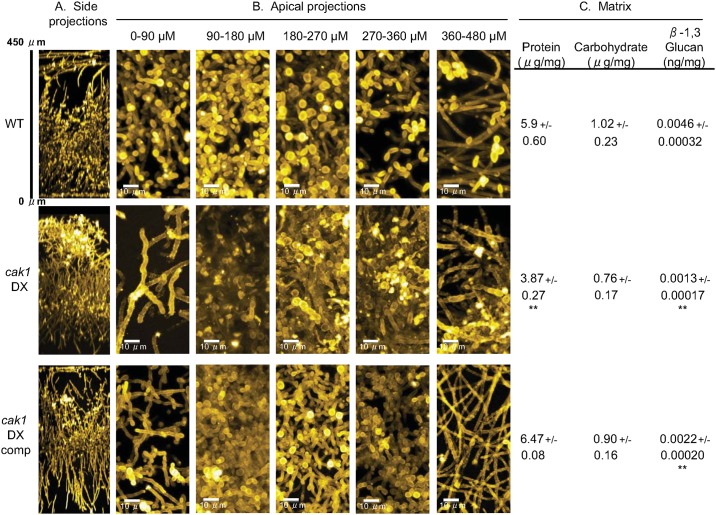
Apical projections of biofilm and measurements of biofilm matrix components. A. Strains were grown under in vitro biofilm conditions for 48 hr, then visualized by confocal microscopy. Side projections of a biofilm of each strain is shown. B. Apical view projections of the same biofilms shown in panel A were obtained using maximum intensity Z-projection of 100 planes at 0.9 μm step-size at the distance indicated from the basal layer. C. Biofilms were grown for matrix isolation. Ten ml of matrix was collected for each strain, biomass collected and dried, and quantitation of matrix total protein, carbohydrate, and normalized values of β-1,3 glucan were determined. Strains: WT (DAY185), *cak1* DX (CW1428), *cak1* DX comp (CW1431). Triplicate samples for all but *cak1* DX carbohydrate (duplicate) determinations. ** = p<0.01 for comparison of either *cak1* DX or *cak1* DX comp to WT.

Biofilms of *C*. *albicans* include both filamentous cells and yeast cells [44, 45]. We sought to determine whether hyperfilamentous DX strains showed altered biofilm formation. For this analysis we focused on the *cak1* DX strain because it had a prominent phenotype. The wild-type, *cak1* DX, and complemented strains all formed biofilms of similar depth, as shown in side-view projections of confocal microscopy images (Fig 4A). However, apical views revealed defects in the *cak1* DX biofilm (Fig 4B). Specifically, cells in the upper regions (above 180 μm from the substrate) displayed aberrant morphology. In addition, cell staining in these regions was uneven, which may reflect cell wall defects. Biofilm growth is accompanied by accumulation of extracellular matrix, a complex mixture of protein, carbohydrate, and other components [46]. We observed that *cak1* DX biofilm matrix had significantly reduced levels of protein (Fig 4C). Levels of one of the best characterized matrix carbohydrate components, soluble β-1,3 glucan, also accumulated at significantly lower levels in the *cak1* DX biofilm than in the wild-type or complemented strain biofilms. These results indicate that diminished Cak1 expression does not prevent biofilm formation, but Cak1 is required for normal biofilm cell morphology and matrix production.

### Relationships between Cak1 and regulators of filamentation and biofilm formation

Results above indicate that several growth-regulatory protein kinases function as negative regulators of filamentation and filamentation-associated gene expression. We sought to determine whether this role of the protein kinases may be mediated by known transcriptional activators of filamentation genes. We again focused on *CAK1* for the analysis.

**Fig 5:**
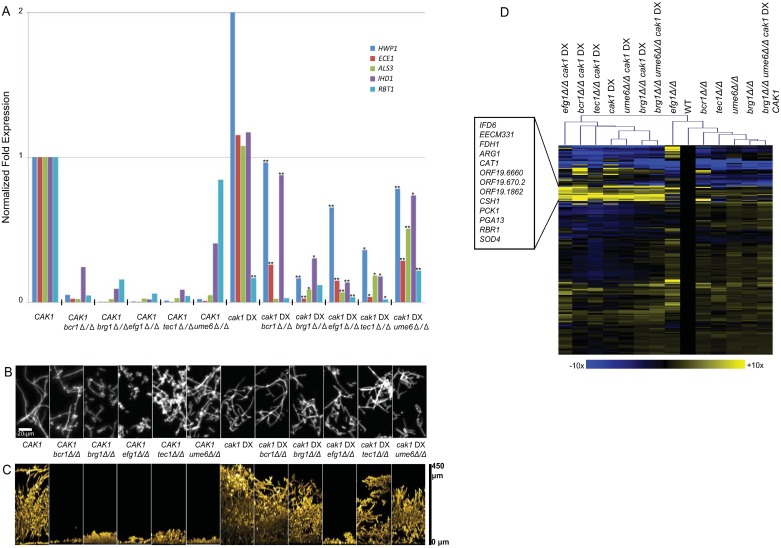
Core filamentation gene expression and phenotypes of filamentation/biofilm activator mutants in *CAK1* and *cak1* DX backgrounds. A. RNA levels for environmental response genes was determined by nanoString for strains grown for 4 hr at 37°C in YPD (S4 Table). Normalized expression levels are shown for core filamentation genes in the strains indicated. Transcription activator mutant strains in a *CAK1* background as well as the *cak1* DX mutant background are shown. Symbols: * = p < 0.05; ** = p < 0.01 for comparison of *CAK1* and *cak1* DX strains carrying the same activator mutation. Strains are listed in S4 Table. B. Cell morphology. Cell cultures were grown for 4 hr at 37°C in YPD, then fixed and stained with Calcofluor White prior to visualization. Strains: WT (DAY185), *bcr1Δ/Δ* (CW1627), *brg1Δ/Δ* (CW1639), *efg1Δ/Δ* (CW1651), *tec1Δ/Δ* (CW1646), *ume6Δ/Δ* (CW1633), *cak1* DX (CW1428), *cak1* DX *bcr1Δ/Δ* (CW1547), *cak1* DX *brg1Δ/Δ* (CW1460), *cak1* DX *efg1Δ/Δ* (CW1603), *cak1* DX *tec1Δ/Δ* (CW1579), *cak1* DX *ume6Δ/Δ* (CW1534). C. In vitro biofilms. Strains were grown under in vitro biofilm conditions for 48 hr, then visualized by confocal microscopy. Cross-sectional views are shown. D. Gene expression profiles. RNA was extracted from cells grown for 4 hr at 37°C in YPD and used for nanoString expression analysis (S4 and S5 Tables). Hierarchal clustering of gene expression data was performed from the average of either three isolates (single and double mutants) or four isolates (triple mutants) using MeV software. Fold change values were obtained by dividing normalized expression values for each strain genotype by the wild type strain (DAY185) for each of the probes. The color scale represents Log2 fold change compared to wild type. (Blue limit: 10-fold down; yellow limit: 10-fold up) See S4 and S5 Tables for strains.

We chose five major activators of filamentation genes: Bcr1, Brg1, Efg1, Tec1, and Ume6. All are required for filamentation-associated gene expression [47–51]. We verified that a panel of newly made mutants affecting these genes had reduced expression of core filamentation genes (Fig 5A, S4 Table). Under these growth conditions (YPD medium at 37°, which is sufficient for induction of the filamentation pathway [52, 53]), filamentous cell morphogenesis was severely defective in the *efg1Δ/Δ* mutant, partially defective in the *brg1Δ/Δ* and *tec1Δ/Δ* mutants, and largely similar to the wild-type strain in the *bcr1Δ/Δ* and *ume6Δ/Δ* mutants (Fig 5B). All of the mutants were also defective in biofilm formation (Fig 5C).

Quantitative measurement of gene expression in single and double mutant strains revealed that each filamentation activator was required for the full gene expression response to reduced *CAK1* levels (Fig 5A). We used filamentation-inducing growth conditions, in which core filamentation gene expression is similar in the *cak1* DX and wild-type strains. In the *cak1* DX strain background, expression of most core filamentation genes assayed was substantially reduced by any filamentation activator deletion mutation. For example, levels of *ECE1* RNA were reduced between 5- and 50-fold by deletion of any single filamentation activator gene in the *cak1* DX background. However, mutation of any particular filamentation activator had less gene expression impact in the *cak1* DX background than in the wild-type *CAK1* background. For example, levels of *ECE1* RNA were increased between 10- and 50-fold by a *cak1* DX alteration in any filamentation activator gene deletion background. Therefore, although the expression of filamentation-associated genes remains largely dependent upon these filamentation activators regardless of the state of *CAK1*, reduced *CAK1* expression causes increased residual filamentation-associated gene expression in the absence of major filamentation activators.

A broader view of gene expression levels supports the idea that the *cak1* DX background is epistatic to filamentation activator defects. This point is illustrated by the fact that the *cak1* DX strain groups together with the *cak1* DX-filamentation activator double mutants by unsupervised hierarchical clustering (Fig 5D). Inspection of the data indicates that the *cak1* DX strain has highly pleiotropic gene expression effects, and many of those effects are insensitive to deletion of filamentation activators. Therefore, our gene expression data indicate that reduced *CAK1* expression can overcome much of the impact of defects in filamentation activators.

Biological impact of the filamentation activator mutations was muted when examined in a *cak1* DX background. For example, biofilm formation ability was restored for all activator mutants except the *efg1Δ/Δ* mutant (Fig 5C). Even though the *efg1Δ/Δ* mutant remained biofilm-defective, its filamentation ability was greatly improved in the *cak1* DX background (Fig 5B). These phenotypic assays indicate that reduced *CAK1* expression can bypass the need for Bcr1, Brg1, Tec1, and Ume6 in biofilm formation, and can overcome the dependence of filamentation on Efg1. These biological results argue that the residual expression of filamentation-associated genes that is observed in filamentation activator mutants in the *cak1* DX background has substantial phenotypic impact.

**Fig 6:**
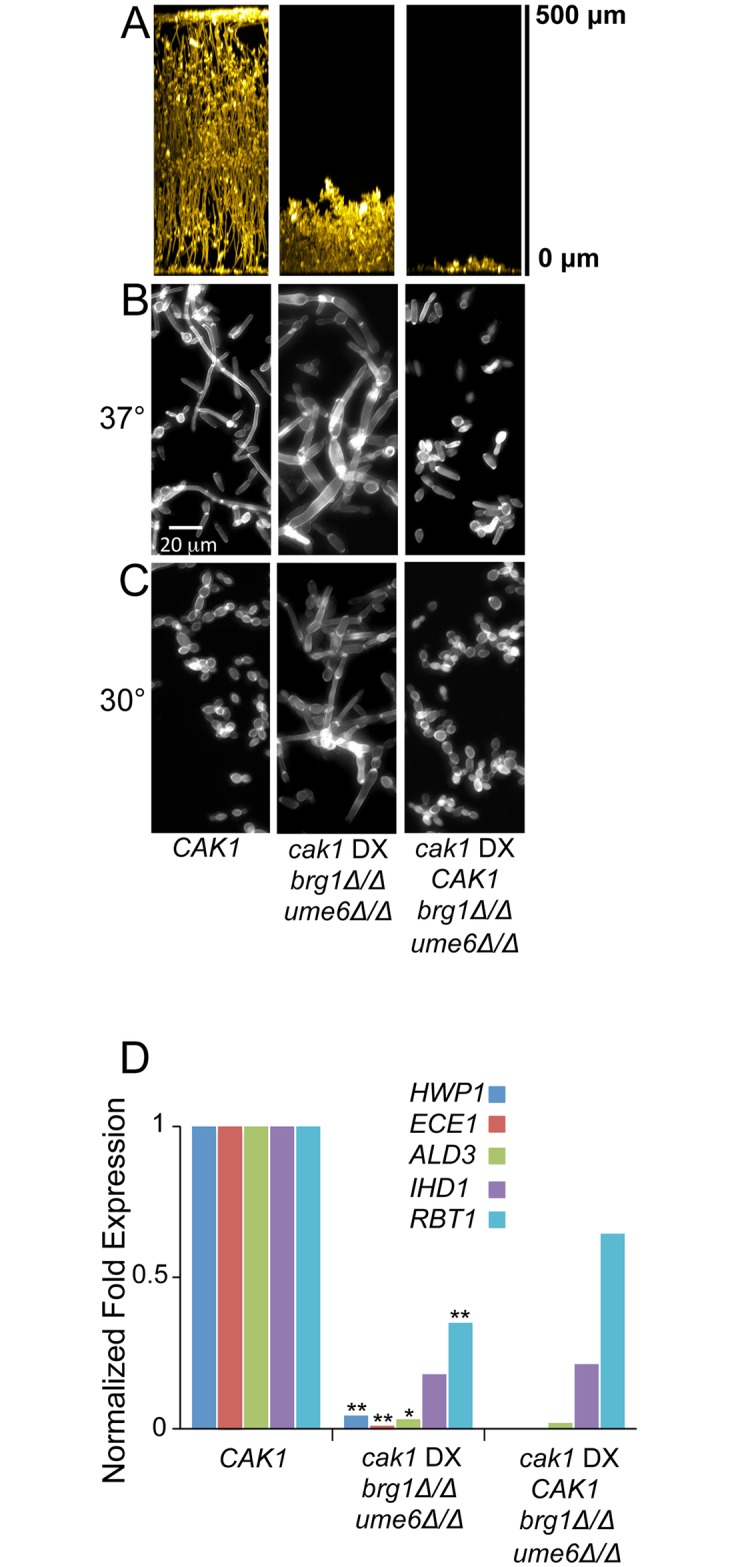
Phenotype and core filamentation gene expression of *cak1* DX *brg1Δ/Δ ume6Δ/Δ* mutant strain. A. In vitro biofilms. Strains were grown under in vitro biofilm conditions for 48 hr, then visualized by confocal microscopy. Cross-sectional views are shown. Strains: *CAK1* (DAY185), *cak1* DX *brg1Δ/Δ ume6Δ/Δ* (CW1715), *cak1* DX *CAK1 brg1Δ/Δ ume6Δ/Δ* (CW1720). B. Cell morphology. Cell cultures were grown for 4 hr at 37°C in YPD, then fixed and stained with Calcofluor White prior to visualization. C. Cell morphology. Cell cultures were grown for 4 hr at 30°C in YPD, then fixed and stained with Calcofluor White prior to visualization. D. RNA levels for environmental response genes were determined by nanoString for strains grown for 4 hr at 37°C in YPD (S5 Table). Normalized expression levels are shown for core filamentation genes in the strains indicated. Symbols: * = p < 0.05; ** = p < 0.01 for comparison of *cak1* DX *brg1Δ/Δ ume6Δ/Δ* to *cak1* DX *CAK1 brg1Δ/Δ ume6Δ/Δ* strains.

The epistasis tests above show that diminished *CAK1* expression can override filamentation/biofilm activator defects, based upon several phenotypic assays. One simple interpretation of these outcomes is that Cak1 acts downstream of the filamentation/biofilm activators [54, 55]. However, each filamentation/biofilm activator's target genes overlap considerably with those of the other filamentation/biofilm activators [48]. This point led us to consider an alternative explanation. Specifically, we considered a model in which diminished *CAK1* expression may stimulate the functions of several filamentation/biofilm activators. A deletion mutation that removes only one activator might be bypassed through the effects on the other activators. We tested this model through analysis of mutant strains with deletions of two filamentation/biofilm activator genes, *BRG1* and *UME6*. Recent studies indicate that Brg1 promotes initiation of filamentation, whereas Ume6 promotes maintenance of filamentation [51, 56]. In addition, our results show that *BRG1* and *UME6* RNA levels are increased in the *cak1* DX background at 30° (S2 and S3 Tables). We observed that the *brg1Δ/Δ ume6Δ/Δ cak1* DX strain had some biofilm formation ability (Fig 6A), and could produce filamentous cells at both 37° and 30° (Fig 6B and 6C). When a wild type copy of *CAK1* was introduced into the strain, biofilm formation was eliminated (Fig 6A) and cell morphology (Fig 6B and 6C) more closely resembled that of the *brg1Δ/Δ* mutant strain (Fig 5B). Quantitative measurement of gene expression in the *brg1Δ/Δ ume6Δ/Δ cak1* DX strain revealed a significant increase in expression of core filamentation genes *HWP1*, *ECE1*, and *ALS3* over that observed in the *CAK1*-complemented strain (Fig 6D), though the magnitude of expression was greatly reduced compared to the *brg1Δ/Δ cak1* DX strain and the *ume6Δ/Δ cak1* DX strain (Fig 5A). The fact that the *cak1* DX phenotypes are less pronounced in a *brg1Δ/Δ ume6Δ/Δ* double mutant background than in either the *brg1Δ/Δ* or *ume6Δ/Δ* single mutant backgrounds is consistent with the hypothesis that both Brg1 and Ume6 contribute to the *cak1* DX phenotypes.

## Discussion

Our results support two main conclusions. First, filamentous morphogenesis is activated together with filamentation-associated gene expression in response to impairment of diverse growth control pathways. These results indicate that growth inhibition can promote *C*. *albicans* biofilm formation and virulence, an idea that has also emerged for bacterial pathogens [57, 58]. Second, activation of the filamentation response by cell cycle/cell growth limitation can have distinct regulatory features from the filamentation response of the wild-type strain. We explore these points in greater detail after a brief discussion of the approach that we have implemented to analyze essential *C*. *albicans* genes.

### Analysis of essential *C*. *albicans* genes

Most studies of *C*. *albicans* essential genes have employed fusions to regulated promoters [12–17]. The GRACE strain collection is an exceptionally useful resource built upon such a platform [12]. The function of a gene is assessed after it is transcriptionally repressed. This approach is extremely useful, but has some limitations. For example, repression is occasionally incomplete [12]. In addition, it can take many generations to dilute out pre-existing gene product after the regulated promoter has been shut off (see references [59] and [60] for examples). Finally, growth under permissive conditions, when the regulated promoter is active, can result in overexpression of the targeted gene [61, 62] and novel phenotypic consequences. Our approach here was to reduce gene expression constitutively, a concept that has proven extremely useful for uniform analysis of *S*. *cerevisiae* essential genes [63]. This approach has the advantage of enabling phenotypic assays under diverse conditions where controlled manipulation of drug concentrations or carbon sources may not be feasible. Also, constitutive down-regulation relieves concern about a protracted time for dilution of pre-existing gene product. Our approach has the disadvantage of potentially selecting for secondary suppressor mutations, and such suppressors may have been the cause of heterogeneity of our *snf1* DX strains. A second disadvantage is some genes seemed to be affected very little by our promoter replacement. These and other considerations lead us to suggest that no single approach is ideal for analysis of all genes under all circumstances.

In fact, it seems advantageous to have several approaches available for gene function analysis. A comparison of DX strain cell morphology with results of prior studies illustrates this point (Table 1). In most cases (7 genes), the DX and prior depletion or deletion strains have the same cell morphology, thus providing validation of results. In two cases–*CDC7* and *CDC28* –the prior conditional depletion strains were filamentous and the DX strains grew as yeast. It seems reasonable that the prior conditional depletion strains had lower gene expression levels than the DX strains. In fact, these two particular DX strains expressed the targeted genes at higher levels than most other DX strains (S1 Table). In the case of *CAK1*, the DX strain was filamentous and the prior conditional depletion strain grew as yeast. We validated the *cak1* DX strain phenotype by both complementation and consistency of independent isolates. Thus it seems reasonable that the DX strain had lower gene expression levels than the prior conditional depletion strain. These examples of gene-specific considerations illustrate that it is useful to have several approaches for functional analysis.

### Relationship between filamentation and cell cycle/cell growth control

Several previous reports show that mutation of cell cycle or cell growth regulatory genes can induce filamentous growth under conditions that are noninducing for wild type strains (reviewed in [19, 20]). A recent genome-scale survey of GRACE strains extended the list of such mutants considerably [18], and our study brings the number of protein kinase-related genes that control cell cycle/cell growth and filamentation from nine to thirteen. Our results together with prior studies indicate that many protein kinases with cell cycle or cell growth regulatory functions are negative regulators of filamentous growth in that their inactivation induces filamentation.

Although the connection between cell cycle/cell growth impairment and filamentous growth is well established, the mechanisms that mediate this relationship are uncertain. Our findings shed light on this connection. Specifically, we found that several major transcriptional activators of filamentation-associated genes are required for the full gene expression response to diminished *CAK1* expression. This result argues that the filamentation response to cell cycle/cell growth impairment probably does not reflect rogue activity of one particular activator, but instead results from activity of signaling pathways that normally govern filamentous growth.

One unexpected outcome of our analysis is that there is substantial expression of filamentation-associated genes in response to diminished *CAK1* levels even in the absence of any key transcriptional activator. The net impact is functional, as revealed through biological assays for filamentation and biofilm formation in vitro. If we interpret the results in the framework of a linear pathway model [54, 55], they suggest that Cak1 may act downstream of the filamentation/biofilm activators to repress target gene expression. However, the overlap among targets of these activators has been well documented [48, 51, 56]. We thus considered the model that diminished *CAK1* expression may stimulate several filamentation/biofilm activators, and that each can compensate partially for the absence of any other. The mechanism of activation would have to be post-transcriptional, because at 37° the *cak1* DX background has little impact on activator RNA levels (S4 Table); several post-transcriptional control mechanisms for filamentation/biofilm activators are known [64–67]. The model that Cak1 has impact on several filamentation/biofilm activators is consistent with our experimental test, which showed that the *cak1* DX phenotype is more strongly affected by absence of Brg1 and Ume6 together than it is by absence of either Brg1 or Ume6 alone. Our results thus argue that the cell cycle impairment by reduced *CAK1* expression results from stimulation of multiple filamentation/biofilm activators.

The relationship between filamentation-associated gene expression levels and biofilm formation ability seems puzzling. For example, expression of filamentation-associated genes is not dramatically higher in the *brg1Δ/Δ ume6Δ/Δ cak1* DX strain, which is biofilm-competent, as compared to the *CAK1* complemented *brg1Δ/Δ ume6Δ/Δ cak1* DX strain, which is biofilm-defective (Fig 6D; S5 Table). Perhaps there is a precise threshold for adhesin gene expression levels that can support biofilm formation. This threshold may be reduced in the *brg1Δ/Δ ume6Δ/Δ cak1* DX strain because it expresses lower levels of *YWP1* than the *CAK1* complemented *brg1Δ/Δ ume6Δ/Δ cak1* DX strain (S5 Table); *YWP1* specifies an anti-adhesin [68]. A second possibility is that reduced *CAK1* activity may cause increased expression of alternative biofilm adhesins. We note that several cell wall protein genes are up-regulated in *cak1* DX strains (Fig 5D); these genes may specify such alternative adhesins.

Our results together with prior studies indicate that it is common for essential protein kinases to function as negative regulators of filamentation and filamentation-associated genes ([18, 31, 32, 35], reviewed in [20]). Other essential genes are also negative regulators of filamentation [18] and one well studied example, *HSP90*, exerts its effects through activities of essential protein kinases [37, 69]. There are a few growth inhibitors that activate filamentation or filamentation-associated genes at sub-lethal concentrations [24, 70–72]. It seems possible that such drugs target some of the essential genes whose inactivation causes increased filamentation, as has been shown for inhibitors of Tor1 and Hsp90 [24, 72].

How may the inverse relationship between cell cycle/cell growth and filamentation benefit *C*. *albicans*? A rationale originally proposed for *S*. *cerevisiae* pseudohyphal growth—that filamentous growth allows escape from an unfavorable environment [73]—seems applicable to *C*. *albicans* as well. A second rationale views this relationship in the context of biofilm formation. The cells at the base of a biofilm are critical for adherence of the entire structure to the substrate. Those cells are likely to be nutrient-limited as well, because they are surrounded by other cells with better access to the outside environment. Filamentous growth forms express key biofilm adhesin genes *ALS3* and *HWP1* [38]. Therefore, induction of filamentation by growth limitation would have the effect of reinforcing biofilm-substrate attachment.

## Methods

### Media

*C*. *albicans* strains were grown at 30°C in YPD or 37°C in YPD, YPD + 10%FBS or in RPMI-1640. Transformants were selected on synthetic medium (2% dextrose, 1.7% Difco yeast nitrogen base with ammonium sulfate and auxotrophic supplements).

### Selection of weakly expressed promoters

Based on data from previous experiments, we searched for genes whose expression was unmodulated between planktonic 30° growth in YPD, 37° growth in hyphal inducing spider media, and in kidney infection models. We then looked among those genes for the ones whose expression was low from nanostring data analysis (normalized expression values below 500 counts) and we selected three of varying expression levels: *PGA5 (ORF19*.*3693)*, *PGA42 (ORF19*.*2907)*, and *ORF19*.*7606* (S6 Table).

### Construction of DX mutants

Heterozygotes were constructed in the BWP17 background by replacing one allele of the gene of interest with the *URA3* marker by homologous recombination. PCR was performed using plasmid pGEM*URA* as template; PCR primers had homology to 100 bp upstream of the ATG or to 100 bp downstream of the stop codon followed by 18–19 bp homology to adaptor sequences on the plasmid surrounding the selective marker. This PCR product was transformed into the *C*. *albicans* strain.

The promoter sequences were inserted downstream of *ARG4* in pRS*ARG4ΔSpe*, oriented in the same direction as *ARG4*, by digesting the plasmid with *Not*I to direct integration of the PCR product carrying promoter sequences by homologous recombination in *S*. *cerevisiae*.

The promoter sequences to *PGA5*, *PGA42*, and *ORF19*.*7606* were inserted into the plasmid. The promoter for *PGA5* included only 400 bp upstream of the start codon (due to the end of an upstream gene located 411 bp upstream of the *PGA5* ORF) and the promoters for *PGA42* and *ORF19*.*7606* each included 950 bp upstream of their respective start codons. In order to be able to use the same primers to amplify each of the promoters from their respective plasmids, the primers used to amplify sequences for the *PGA42* and the *ORF19*.*7606* promoters for insertion into the plasmid included homology to the 30 nucleotides directly upstream of the start codon of *PGA5* as a sequence adaptor substituting for those equivalently located nucleotides of *PGA42* and *ORF19*.*7606*. Thus the forward and reverse primers for amplifying promoter sequences from pRS*ARG*-DX1 (*PGA5* promoter), pRS*ARG*-DX2 (*PGA42* promoter), or pRS*ARG*-DX3 (*ORF19*.*7606* promoter) were the same for each of the promoters for any specific gene.

The primers for amplifying promoter sequences to be transplaced in front of specific genes were designed to delete somewhere between 50 and 500 bp of the native promoter region of the gene. The general design of the primers used for amplifying the promoter sequences to be transplaced in front of genes is as follows:

Forward primer:

5’ [100 bp upstream sequence of *GENE X* within 500 bp of ATG] [GTGTGGAATTGTGAGCGGATA (the reverse complement of bp 3960–3980 pRS*ARG4ΔSpe* -this sequence is upstream of *ARG4*)]3’

Reverse primer:

5’[reverse complement of 1^st^ 100 nts of *GENE X* ORF] [GATGGATTAAGATGATTGATTGTGATGATT (the reverse complement of the 30 bp adaptor sequence for promoters from 30 bp upstream of *PGA5* orf to 1 bp upstream of *PGA5* orf)] 3’

Primer pairs were designed to check the specific alleles present in any given transformant: an upstream F check and an ORF rev check to check for the presence of a wild type allele of a gene; the upstream F check and a *URA3* rev check to check for the *URA3* marked allele; and an adaptor seq F check and the ORF rev check to check for the promotor transplacement.

Strains used are listed in S7 Table.

### Complemented strains

When complementing DX mutants, the WT allele was amplified from genomic DNA (SC5314), including 1000 bp upstream and 250 bp downstream of the ORF (shorter distances were used when there were additional genes located within this region). Complementation primers were approximately 80 bp in length and were comprised of a sequence to direct *in vivo* recombination into plasmid pDDB78 (CAATTTCACACAGGAAACAGCTATGACCATGATTACGCCAAGCT for the forward primer and GTCGACCATATGGGAGAGCTCCCAACGCGTTGGATGCATAG for the reverse primer) followed by a 45mer gene specific sequence. The complementing PCR product was co-transformed along with *EcoR*I/*Not*I digested pDDB78 into the *S*. *cerevisiae* BJ2698 strain (*his1*). The resulting complementing plasmid was amplified in *E*. *coli* and digested with *Nru*I to direct insertion to the *his1* locus of the DX mutant strains. *Nru*I digested pDDB78 was inserted into mutant strains to obtain a marker-matched prototrophic mutant.

### Double mutants

When DX mutations were in conjunction with a homozygous deletion of another gene, an unmarked deletion was first constructed of the other gene using the *URA3* mini-blaster protocol [74] which utilizes a recyclable cassette, and then this strain was used as the parent for the construction of the DX mutant. His^+^ prototrophs were made by inserting *Nru*I digested pDDB78 into the mutant strains.

### Triple mutants

An unmarked deletion of *BRG1* was first constructed using the *URA3* mini-blaster protocol [74]. Then *UME6* was deleted using a transient CRISPR-Cas9 system [75, 76] utilizing a *NAT1* marker. This strain was used as the parent for the construction of the *cak1* DX mutant. His+ prototrophs or *CAK1* complemented strains were constructed as described above.

### RNA extraction

Overnight cultures of cells were diluted into 50 mls fresh medium at specified OD. Cultures were allowed to grow with shaking and cells were then harvested by vacuum filtration and quickly frozen at -80°C until RNA extraction.

RNA extractions were performed using Qiagen RNeasy Mini Kit (cat#74104) with the following modifications. Cells were resuspended from the membranes with 2 x 900 μl ice cold water washes with vortexing for 30 sec after each addition. 1.5 mls of cell suspension was added to prechilled microfuge tubes and centrifuged at top speed for 30 sec. 600 μl RLT + 1%BMSH was added to resuspend the cells and this was added to a fresh 2 ml screw cap tube containing 300 μl Zirconia beads (Ambion, Fisher Scientific) and 600 μl phenol:chloroform:isoamyl alcohol 25:24:1, vortexed on a mini-beadbeater (Biospec Products) for 3 min, and centrifuged at 14000 rpm for 5 min at 4°C. 550 μl of the aqueous phase was transferred to a new microfuge tube, an equal volume of 70% ethanol added, and RNA isolation proceeded as the manufacturer’s instructions.

### NanoString analysis

NanoString analysis is a sensitive method for analyzing gene expression of 100–800 targets at a time. For our analysis, 166 targets associated with environmental response pathways, including cell wall stress, osmotic and oxidative stress conditions, hypha development and biofilm formation, along with 15 targets corresponding to the mutants constructed for this work, were selected for analysis. For each nanoString assay, 125–300 ng of *Candida* RNA extracted from an in vitro liquid culture was added to a nanoString codeset mix and incubated at 65°C overnight (16–18 hours). The reaction mixes were loaded on the nanoString nCounter Prep Station for binding and washing, and the resultant cartridge was transferred to the nanoString nCounter digital analyzer for scanning and data collection. A total of 600 fields were captured per sample. To calculate gene expression ratios among different samples, we normalized adjusted raw counts by total counts of 181 genes after background subtraction [77]. The heat maps were generated using Multiexperimental Viewer 4.9.0 [78]. Preliminary determination of expression levels of the DX alleles was carried out on single his^-^ isolates. Statistical significance was assayed by t-test.

### Cell microscopy

Strains were grown overnight and diluted into fresh 30° or 37° YPD at an OD_600_ of 0.1. Cultures were grown for 4 hr and cells pelleted. 0.5 mls of media was retained in the tube, and cells transferred to microfuge tubes. Cells were pelleted and the supernatant removed. One ml 4% formaldehyde/PBS was added and the tubes were vigorously vortexed for 15 min. Cells were washed one time with PBS, resuspended in 100 μl PBS, and then vortexed. Calcofluor White (5.5mg/ml in 50% DMSO) was added to 0.2 mg/ml, cells were vortexed and then incubated for 15 min with an occasional flicking. Cells were stored at 4° in the dark until visualization. Cells were diluted 1:2 with PBS and pipetted onto slides coated with concanavalin A. Cells were visualized with a Zeiss Axio Observer Z.1 fluorescence microscope and a 20x objective.

### Biofilm microscopy

The strains being analyzed were grown overnight in YPD at 30°C. The overnight cultures were used to inoculate wells with 2 mls of fresh YPD media at an OD_600_ of 0.5 on silicone squares (Bentec Medical Inc.) that were pretreated with fetal bovine serum (FBS). The cells were allowed to adhere to the silicone for 90 min in an incubator-shaker at 37°C and 60 rpm. Following the adherence, the squares were washed in PBS to remove any nonadherent cells and placed in wells containing 2 mls of new YPD media. Biofilm imaging is adapted from [79] with modifications. After 48 hours, the biofilms were fixed using 4% formaldehyde and 1.5% glutaraldehyde in 1xPBS on an orbital mixer for 1 hour. After fixation the specimens were washed in 1xPBS. The fixed biofilms were stained with concanavalin A, Alexa Fluor 594 Conjugate (Life Technologies) at a concentration of 25 μg/ml in PBS for two days on an orbital mixer. The fixed and stained biofilms on the silicone squares were then transferred to glass scintillation vials. To dehydrate the samples, 2 mls of methanol were added to the samples and allowed to infiltrate on an orbital mixer for 20 min. The methanol was aspirated out and 2 mls of methanol were briefly added. After the 100% methanol addition, a 50:50 mixture of methanol and methyl salicylate was added. The 50:50 mixture was aspirated out and replaced with 100% methyl salicylate. The vials were gently agitated until the samples were completely cleared through the matching of refractive index. In order to image these samples using an inverted confocal microscope and avoiding the use of plastic, a cover glass was cemented to the bottom of a black-anodized aluminum stage insert. A silicone ring (thickness 300μm) and a small amount of methyl salicylate were added to this constructed well and the biofilm was placed on the ring with the apical side facing down, using the surface tension between the methyl salicylate and the silicone square to hold the silicon in place. The biofilms were imaged using a slit-scan confocal optical unit on a Zeiss Axiovert 200 microscope. A 40x 0.85-numerical aperture oil immersion objective was used in order to provide enough working distance to focus through the full thickness of the biofilms. Optical sections were collected in several series of 130 planes with a total sum of 500 planes (Fig 4) or 557 planes (Fig 6A) at 0.9 μm step-size. The stacks were concatenated and processed using FIJI software [80]. The images were processed using the Background Subtract plugin and the final images were obtained using a resliced, maximum intensity Z-projection. The apical view projections were obtained using the Temporal Color-code plugin and the Ice lookup table.

### Cek1 phosphorylation analysis

Protein extraction and immunoblots were performed as described [81]. Proteins were extracted from cells grown to mid-log phase (~5 hr) at 30°C in YPD media. Protein samples were separated on 10% SDS polyacrylamide gels (SDS-PAGE) and transferred to nitrocellulose membranes (Protran BA85, VWR International Inc., Bridgeport NJ). Membranes were blocked in immunoblot buffer (5% nonfat dry milk, 10mM Tris-HCl [pH 8], 150mM NaCl and 0.05% Tween 20) for 16h at 4°C. Cdc2 p34 antibody that recognizes the PSTAIRE motif was used as a loading control (Santa Cruz Biotechnology, Santa Cruz, CA; #sc-53). P~Cek1 was detected by p42/p44 antibodies (Cell Signaling Technology, Danvers, MA; #4370). Secondary antibodies included goat α-mouse IgG–HRP (Bio-Rad Laboratories, Hercules, CA; #170–6516), goat α-rabbit IgG-HRP (Jackson ImmunoResearch Laboratories, Inc., West Grove, PA; #111-035-144), donkey α-goat IgG-HRP (Santa Cruz Biotechnology, Santa Cruz, CA; #sc-2020). Ponceau S (Sigma, St. Louis, MO; #P7170) was used to confirm protein levels. WesternBright MCF fluorescent Western blotting kit from Advansta Inc. (Menlo Park, CA; LPS #K-12045-D20) was used for detection. Densitometry was performed on immunoblots to calculate the levels of P~Cek1 in selected DX mutants. After background subtraction, band intensity of P~Cek1 and PSTAIRE were measured for each sample by ImageJ (https://imagej.nih.gov/ij/). The ratio of P~Cek1/PSTAIRE was set to 1.0 for wild type and calculated for other samples to measure the relative difference in P~Cek1 levels.

### Biofilm production

Extracellular matrix was collected from two roller bottles, as published previously [82]. Briefly, cells from an overnight culture were used to create an inoculum of 10^6^ cells/ml in RPMI-MOPS. One ml inoculum was added to each bottle, and biofilms were incubated at 37°C for 48 hr at 50 rpm, with a media exchange at 24 hr. The biofilms were removed from the bottles using a spatula and suspended in water, then sonicated for 20 min. These were centrifuged at 4,000 rpm for 20 min at 4°C, separating the soluble matrix from the cell pellet (biomass). For normalization of subsequent ELISA data, the biomass of each biofilm from the group was collected, lyophilized, and weighed.

### BCA protein assay and total carbohydrate

Biofilm matrix protein was measured using a BCA protein assay (Thermo-scientific), slightly modified from the manufacturer’s instructions. Briefly, 100μl of isolated matrix samples in triplicate were incubated with the BCA reagents 30min at 37°C. ODs were read at 562nm and protein concentrations calculated from a BSA standard curve. Raw data generated by the BCA assay was normalized by relative biofilm biomass then presented as μg/mg. The total carbohydrate contents of each sample was measured as hexoses by the phenol-sulfuric acid method and normalized by biomass. T-tests were used to determine statistical significance for both BCA and phenol-sulfuric acid assays.

### ELISA

Matrix samples were analyzed by ELISA using a monoclonal antibody against β-1,3 Glucan (Australia Biosupplies) as previously described [46, 83]. This assay used laminarin (Sigma) as standard curve, at least two biological replicates were performed, and the mean of three technical replicates from one representative assay was calculated. Once normalized by biomass, these values were presented as μg/mg. T-tests were used to determine statistical significance.

## Supporting Information

S1 Fig Gene expression profiles of single isolates of DX mutants. RNA was extracted from cells grown for 4 hr at 30°C in YPD or in addition for the WT strain, grown for 5 hr at 37°C in RPMI and used for nanoString expression analysis (S2 Table). Hierarchal clustering of gene expression data was performed from single isolates using MeV software. Fold change values were obtained by dividing normalized expression values for each mutant strain by the wild type strain (DAY286) for each of the probes. The color scale represents Log2 fold change compared to wild type. (Blue limit: 10-fold down; yellow limit: 10-fold up) See S2 Table for strains.
(PPTX)

S1 Table DX allele RNA levels. NanoString measurements of DX allele expression are provided as normalized numbers following RNA extraction from cells grown for 4hr at 30°C in YPD or 5 hr at 37°C in RPMI.
(DOCX)

S2 Table NanoString expression data for DX mutants. NanoString measurements of target gene expression are provided as normalized numbers following RNA extraction from cells grown for 4hr at 30°C in YPD. WT, in addition, was also analyzed following growth for 5 hr 37°C in RPMI.
(XLSX)

S3 Table NanoString expression data for independent isolates of DX mutants and complemented strains. NanoString measurements of target gene expression are provided as normalized numbers following RNA extraction from cells grown for 4hr at 30°C in YPD.
(XLSX)

S4 Table NanoString expression data for *cak1* DX filamentation activator double mutants. NanoString measurements of target gene expression are provided as normalized numbers following RNA extraction from cells grown for 4hr at 37°C in YPD.
(XLSX)

S5 Table NanoString expression data for *cak1* DX *brg1Δ/Δ ume6Δ/Δ* mutants. NanoString measurements of target gene expression are provided as normalized numbers following RNA extraction from cells grown for 4hr at 37°C in YPD.
(XLS)

S6 Table Gene expression levels of *PGA5*, *PGA42*, and *ORF19*.*7606*. NanoString measurements of target gene expression are provided as normalized numbers following RNA extraction from *Candida albicans* harvested from the listed growth conditions.
(XLSX)

S7 Table List of yeast strains used in this study. (XLSX)
