# Supplementary material for: Bypass of Candida albicans Filamentation/Biofilm Regulators through Diminished Expression of Protein Kinase Cak1
Source: PLoS Genet. 2016 Dec 9;12(12):e1006487. doi: 10.1371/journal.pgen.1006487 (PMC5147786; doi:10.1371/journal.pgen.1006487)
Supplement: S1 Fig — RNA was extracted from cells grown for 4 hr at 30°C in YPD or in addition for the WT strain, grown for 5 hr at 37°C in RPMI and used for nanoString expression analysis (S2 Table). Hierarchal clustering of gene expression data was performed from single isolates using MeV software. Fold change values were obtained by dividing normalized expression values for each mutant strain by the wild type strain (DAY286) for each of the probes. The color scale represents Log2 fold change compared to wild type. (Blue limit: 10-fold down; yellow limit: 10-fold up) See S2 Table for strains. (PPTX) [file pgen.1006487.s001.pptx]

## Slide 1
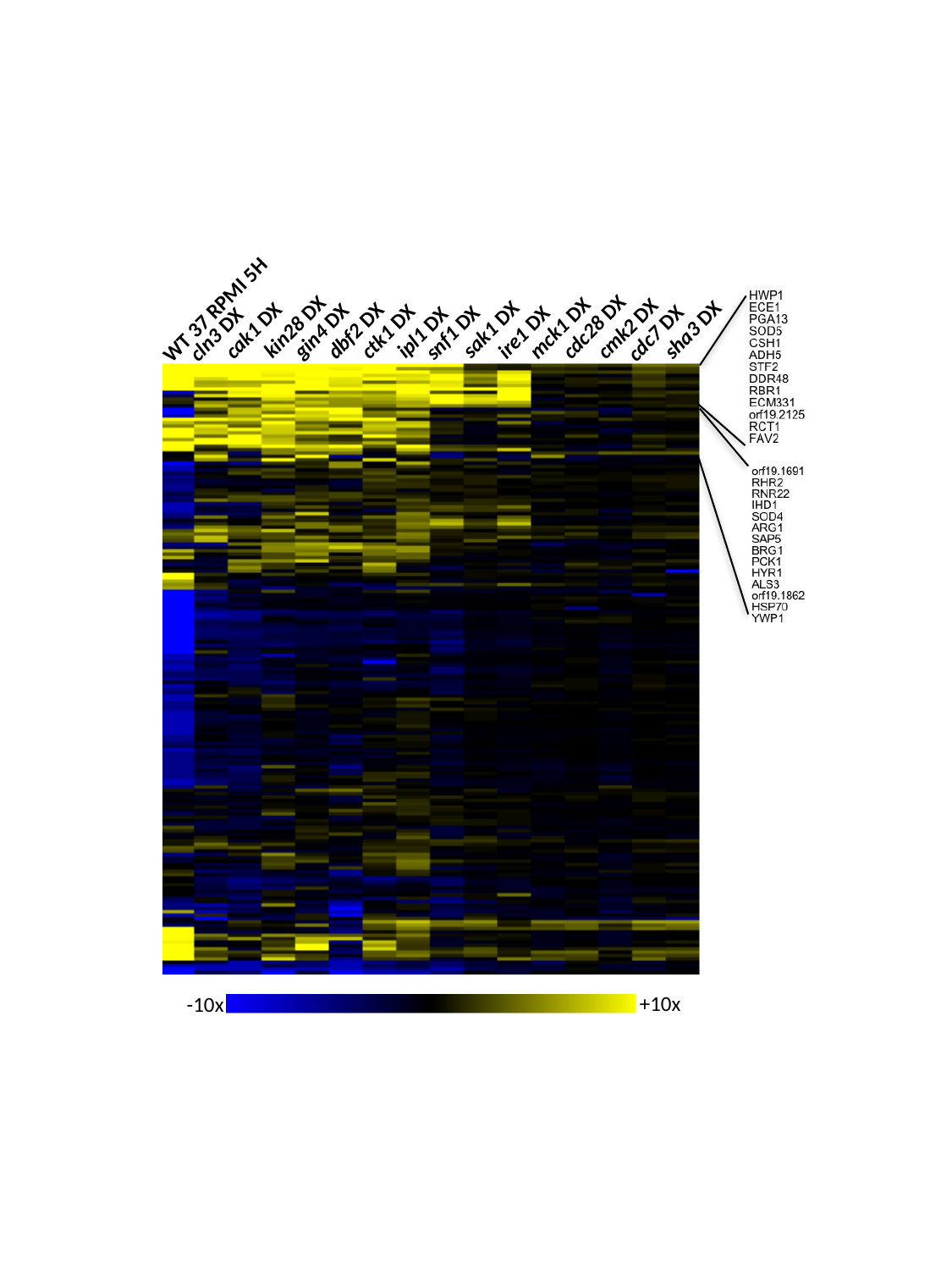

WT 37 RPMI 5H
snf1 DX
cdc7 DX
cln3 DX
kin28 DX
cdc28 DX
mck1 DX
cmk2 DX
sak1 DX
sha3 DX
cak1 DX
dbf2 DX
gin4 DX
ctk1 DX
ire1 DX
ipl1 DX
+10x
-10x
