# Supplementary material for: Bypass of Candida albicans Filamentation/Biofilm Regulators through Diminished Expression of Protein Kinase Cak1
Source: PLoS Genet. 2016 Dec 9;12(12):e1006487. doi: 10.1371/journal.pgen.1006487 (PMC5147786; doi:10.1371/journal.pgen.1006487)
Supplement: S1 Table — NanoString measurements of DX allele expression are provided as normalized numbers following RNA extraction from cells grown for 4hr at 30°C in YPD or 5 hr at 37°C in RPMI. (DOCX) [file pgen.1006487.s002.docx]

| Promoter |  | Percent of WT  Expression^1^ | Counts in  WT strain | Counts in  mutant strain | Percent of WT  Expression^1^ | Counts in  WT strain | Counts in  mutant strain |
| --- | --- | --- | --- | --- | --- | --- | --- |
|  |  | 30° | 30° | 30° | 37° + serum | 37° + serum | 37° + serum |
| *PGA5* | *cak1* | 2 | 2254 | 37 | 2 | 791 | 14 |
|  | *cdc7* | 18 | 2231 | 409 | 27 | 787 | 214 |
|  | *cdc28* | 28 | 3632 | 1011 | 29 | 1019 | 300 |
|  | *cln3* | 1 | 4354 | 26 | 2 | 962 | 17 |
|  | *cmk2* | 4 | 2189 | 90 | 2 | 1025 | 22 |
|  | *ctk1* | 5 | 1374 | 69 | 11 | 493 | 54 |
|  | *gin4* | 2 | 4192 | 84 | 1 | 1278 | 11 |
|  | *ire1* | 5 | 1617 | 77 | 21 | 674 | 141 |
|  | *mck1* | 4 | 2357 | 83 | 2 | 880 | 22 |
|  | *sak1* | 4 | 1962 | 79 | 2 | 777 | 16 |
|  | *sha3* | 12 | 1304 | 160 | 1 | 5312 | 38 |
|  | *snf1* | 3 | 2079 | 59 | 2 | 891 | 22 |
|  |  |  |  |  |  |  |  |
| *PGA42* | *dbf2* | 9 | 2347 | 216 | 18 | 783 | 137 |
|  |  |  |  |  |  |  |  |
| *ORF19.7606* | *ipl1* | 80 | 821 | 658 | 119 | 281 | 335 |
|  | *kin28* | 16 | 984 | 157 | 28 | 363 | 100 |

**S1 Table. DX mutant RNA levels.**

1. All expression levels are compared to DAY286 (WT) grown in the same condition. RNA was extracted from strains grown either at 30° 4hr YPD or 37° 5hr YPD + 10% FBS and analyzed on the nanoString platform.
